# Palliative care and the arts: vehicles to introduce medical students to patient-centred decision-making and the art of caring

**DOI:** 10.1186/s12909-017-1098-6

**Published:** 2017-12-16

**Authors:** Carlos Centeno, Carole Robinson, Antonio Noguera-Tejedor, María Arantzamendi, Fernando Echarri, José Pereira

**Affiliations:** 1 Faculty of Medicine, University of Navarra, Pamplona, Navarra Spain; 2 ATLANTES Research Programme, Institute for Culture and Society, University of Navarra, Edificio Bibliotecas, Campus Universitario, 31009 Pamplona, Spain; 3 IdiSNA, Pamplona, Spain; 4 Faculty of Health and Social Development, School of Nursing, University of British Columbia, Okanagan, Vancouver, Canada; 5 Area Educational, University of Navarra Museum, Pamplona, Navarra Spain; 6 Department of Family Medicine, University of Ottawa, Hamilton, Canada; 7 Division of Palliative Medicine, Department of Family Medicine, McMaster University, Ottawa, Canada

**Keywords:** Medical education, Palliative care, Arts

## Abstract

**Background:**

Medical Schools are challenged to improve palliative care education and to find ways to introduce and nurture attitudes and behaviours such as empathy, patient-centred care and wholistic care. This paper describes the curriculum and evaluation results of a unique course centred on palliative care decision-making but aimed at introducing these other important competencies as well.

**Methods:**

The 20 h-long optional course, presented in an art museum, combined different learning methods, including reflections on art, case studies, didactic sessions, personal experiences of faculty, reflective trigger videos and group discussions. A mixed methods approach was used to evaluate the course, including a) a post-course reflective exercise; b) a standardized evaluation form used by the University for all courses; and c) a focus group.

**Results:**

Twenty students (2nd to 6th years) participated. The course was rated highly by the students. Their understanding of palliative care changed and misconceptions were dispelled. They came to appreciate the multifaceted nature of decision-making in the palliative care setting and the need to individualize care plans. Moreover, the course resulted in a re-conceptualization of relationships with patients and families, as well as their role as future physicians.

**Conclusions:**

Palliative care decision-making therefore, augmented by the visual arts, can serve as a vehicle to address several competencies, including the introduction of competencies related to being patient-centred and empathic.

## Background

The practice of medicine requires more than interviewing and examining a patient, ordering appropriate investigations, making the right diagnosis and initiating a treatment plan. While these skills are essential as is knowledge of sciences such as anatomy, pharmacology, and biochemistry, they are insufficient to make an excellent clinician.

These skills need to be augmented by a set of attributes, attitudes and behaviours that differentiate a mediocre or even good doctor from an excellent one. Being empathic, patient-centred, compassionate, humble and respectful are essential components of being a whole physician able to provide wholistic care.

Medical Schools are challenged to find ways to catalyze and nurture these attributes and behaviours. The task is made even more challenging given evidence that altruism and empathy decline over the course of medical school and specialty training [1, 2]. Increasing cynicism and detachment may partly account for this and hidden curricula that do not make these attributes explicit and valued across the whole medical school or residency learning experience aggravate the problem [3, 4].

A number of strategies and initiatives to reverse this and to strengthen patient-centredness, empathy, compassion and whole-person care amongst medical students and residents (registrars) have been reported. The Harvard University in Boston, for example, implemented an undergraduate curriculum that was designed to heighten humanitarianism [5]. Zazulak and colleagues used an arts-based programme to nurture the affective and cognitive components of empathic development [6].

The University of Navarra in Spain values the attributes of empathy, compassion and caring that enhances human dignity and is striving to make these overt throughout its medical curricula. It is also working to strengthen the palliative care (PC) related content in those curricula given the emergence of Palliative care education as a priority area in medical undergraduate and postgraduate education [7–9]. A recent World Health Organization (WHO) recommendation, for examples, states that basic training in PC should be integrated as a routine element of all undergraduate medical education [10].

With these priorities in mind, the University of Navarra has been seeking appealing ways to engage its learners in these areas. We posit that palliative care provides an excellent platform and vehicle to nurture empathy, compassion, patient-centredness and whole person care. By its very definition and practice, the patient is viewed as a unique individual and person. It requires considering where the patient is coming from and what the patient and his family and loved ones are experiencing. This is particularly true during the decision-making process where many different factors need to be considered Two patients with advanced disease and with the same diagnosis and burden of disease may make very different decisions when faced with a complication such as pneumonia or deciding on whether to pursue third- or fourth-line chemotherapy.

Moreover, palliative care also provides a platform to support other important competencies such as interprofessionalism, communication, and sensitivity to different cultures, and religious and spiritual needs and viewpoints.

In 2005 The University of Navarra in Pamplona, Spain, started integrating palliative care in its six-year medical school curriculum by way of an optional course in the 6th year. In 2011 the course was made compulsory for all students in their sixth (clinical) year [11]. While these represented good initial steps, a need was identified to complement the final-year course with an introduction to palliative care and the related competencies its supports (particularly empathy, patient-centeredness and decision-making) earlier in the curriculum, including the pre-clinical years. This paper describes a pilot course that used palliative care, end-of-life decision-making and the arts to introduce medical students earlier to wholistic patient care. This piece is part of the ATLANTES Research Program of the Institute for Culture and Society (ICS) of the University of Navarra, where the message of Palliative Care is researched with a focus from the humanities and social sciences.

## Methods

### The intervention: Curriculum

The curriculum team (CC, JP, AN and MA) felt that the visual arts warranted exploration as a potential catalyst for some of the learning objectives, particularly those related to keeping an open mind, self-awareness and appreciating the different perspectives that make every situation and patient experience unique [6, 12–15].

A decision was therefore made to host the course in the University’s new modern art museum. In addition to the artwork, the gallery offered a quiet, reflective ambience and an architecture that underscored how the same thing may look very different from different viewing angles. Each day started with a thirty to forty minute- long introduction to pre-selected artworks (paintings, photographs, sculptures or stand-alone displays) by a gallery curator. Students and faculty were asked to reflect on what they saw in each piece and what it represented for them, and what their emotional responses to each work were. A facilitated large group discussion followed. Faculty provided clinical examples from their own work experiences that connected these discussions with real life to underscore the clinical relevance of the exercise.

After this daily reflection, learners made their way to a museum classroom. Several learning methods were used in the classroom, including case-based small and large group discussions and didactic overviews of the topics at hand. Faculty members were specifically tasked to share their real-life stories and examples. Short trigger videos highlighting an issue or communication scenario were also used. These included trigger Snippets and Doodles from Pallium Canada and material from Pallium Canada’s Learning Essential Approaches to Palliative Care (LEAP) courseware. The course program is summarized in Table 1.

**Table 1:** The Course Programme

DAY	HOURS		Topics	
		Part 1	Part 2	Part 3
Day 1	3 h	• Introductions and Course Orientation Reflections on museum art works	• The Illness Experience	• Defining Palliative Care
Day 2	3 h	• Reflections on museum art works	• Suffering • Decision-making frameworks and ethical issues	• Hydration at the End of Life • Nutrition at the End of Life
Day 3	3 h	• Reflections on museum art works	• Dignity & hope • Empathy	• Principles of Pain Management • Psychological and Spiritual/Religious Care
Day 4	3 h	• Reflections on museum art works	• Being Aware: connecting with our feelings, viewpoints and biases	• Respiratory Symptoms • Delirium • Last days and hours
Day 5	3 h	• Reflections on museum art works	• Society and Death and Dying	• Desire for death
Day 6	2 h		• An Introduction to Essential conversations (videos)	• Post course reflection and course evaluation

An interprofessional faculty team made up of doctors, a nurse, a sociologist, and a psychologist/ethicist) was deliberately chosen so as to model interprofessional collaboration and the different perspectives that each professional brings and how these various perspectives and competencies contribute to wholistic care.

To connect the course with their other 2nd-year topics, opportunities were also sought to highlight the relevance of their other current topics in the decision-making process. These included pharmacology (e.g. mechanism of action and metabolism), anatomy (e.g. pain pathways) and biochemistry (e.g. inflammatory mediators and cachexia).

Given the unique nature of the course and the need to first test the concept before broader implementation, it was decided that it would be an optional course that would be presented after the end of the academic year to volunteers who were interested and available; classes were held in the evening from 4 pm to 7 pm. The team recognized that this approach would likely result in the recruitment of a highly motivated, self-selected group of learners.

Although the course was first intended for 2nd-year students, students in other years also expressed interest. This presented a design challenge as various learning needs had to be accommodated. The experiences of the more senior students (5th and 6th Years), particularly their observations and their clinical experiences, were harnessed and they were encouraged to share these during the course. They were also asked to respond to some of the junior students’ questions related to pharmacology and pathophysiology, thereby providing opportunities for them to review these and also to start serving as role models to their junior colleagues.

The course was presented within the school’s International Stream in which courses, particularly in the pre-clinical years, are presented in English. The students receive 3 ECTS credits for the course.

### Evaluation framework

A mixed methods approach was used to evaluate the learning experience, its impact and the various methods used in course. This consisted of a) a reflective exercise in which students were invited to write short responses to three open-ended questions/topics (See Table 2); b) a standardized form used by the University for students to evaluate all the courses they attend (made up largely of a Likert-type 0 to 5 point scales); and c) a focus group (voluntary) of students. Faculty also did a debriefing exercise to share their thoughts and experiences. Participation in b), and c) required students to sign an informed consent.

**Table 2:**
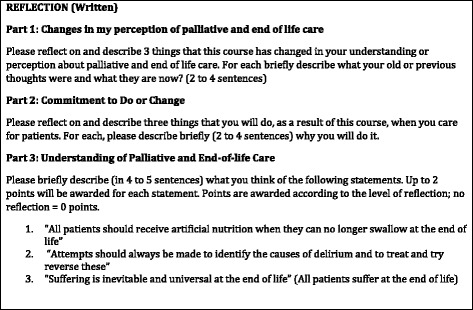
Reflection questions for students post course

We were curious about whether having the course in the museum and integrating art made a difference to the learning experience. Since this had not been previously assessed, all students were invited to participate in a one-hour focus group, eight months later, to explore this question led by a researcher who was not involved in the course design and its delivery (CR). Eight students participated and all agreed to have the focus group video-recorded. In addition, four students spontaneously commented on the usefulness of art in their written responses to the questions above.

### Data analysis

The students’ written responses were in English (9) and Spanish (11). In order to fully understand the Spanish responses and inform analysis, one of the authors (AN) made a culturally appropriate translation with CR.

The open-ended questions and focus group data were analysed using constant comparison to develop an interpretive description (Thorne method) [16]. This is an inductive analytic approach designed to create ways of understanding clinical and learning phenomena that yield relevant practical knowledge. The identified themes are presented including pertinent quotes.

The responses to the quantitative survey (standardized course evaluation) were analysed and compared to other courses in the curriculum. Student identifiers were removed from the written submissions to ensure anonymity.

The evaluation component of the course received approval from the Research Ethic Committee of the University of Navarra (project 120/2015). Students were informed of the study and those interested in participating signed consent. Lack of consent to participate in the evaluation component did not disqualify them from participating in the course.

## Results

A total of 20 medical students (aged 18 to 22 years) participated in the course; 8 were in the 2nd year, 2 in the 3rd year, 3 in the 4th year, 6 in the 5th year, and 1 in the 6th year of study. Nineteen were women. All completed the standardized evaluation of the course provided by the Faculty. Eight accepted the invitation for the focus group, conducted 6 months after the course.

### Standardized course evaluation

The results of the standardized course evaluation by the learners are summarized in Table 3. Students confirmed the relevance of the course content (mean 5/5) and the course was rated very positively across a number of parameters. The course also received high scores relative to other courses that year.

**Table 3:** Evaluation of the Palliative Care Decision Making course and comparisons with other courses offered the same year (Original survey is in Spanish; 0 to 5 scales are used- the higher the score the more positive the evaluation positive)

COURSE	This course (Palliative Care Decision Making)	Other optional courses	All subjects Faculty of Medicine
Surveys received (total asked) Respone ratio (%)	20 (20) 100%	139 (212) 65%	6.409 (11.758) 54%
	Mean (*)	Mean (*)	Mean (*)
The classes were well prepared	4,6	4,7	4,1
Recommended literature and other materials were useful	4,4	4,0	3,5
The faculty roused our interest in the course.	4,9	4,6	3,5
Active participation by students was encouraged	5,0	4,6	3,4
The faculty used appropriate learning methods	4,6	4,6	3,8
The course learning objectives were clear.	4,6	4,3	3,7
The Faculty were open to addressingquestions.	5,0	4,8	4,1
The Faculty was professional with the learners.	5,0	4,9	4,3
The evaluation criteria of the course were clear.	3,4	4,1	3,7
The evaluation criteria were appropriate.	4,1	4,3	3,6
The faculty enhanced my learning.	5,0	4,7	3,7
In this course I learned things that are valuable for my university education	5,0	4,6	3,9
The method of teaching/facilitating enhanced my my attendance in the course.	5,0	4,5	3,4
The learning activities have helped me improve other skills (such as oral and written communication skills, teamwork, use of information,and critical appraisal).	4,9	4,4	3,2
Overall, I am very satisfied with the course [as a learning experience]	4,9	4,6	3,5
Overall total	4,7	4,5	3,7

(*) Only means are shown as only aggregate data (no original data) were provided to the course team; secondary analysis was not possible. For brevity, not all ítems are shown but all ítems that scored less than other courses are shown

### Interpretive description of the students’ written and focus group responses

#### What did the students learn and how will this influence their practice?

The students wrote in English (9) or Spanish (11). Their responses reflect changes in thinking, feeling, and doing. Their responses were remarkably consistent across the 20 students.

Prior to the course, the students shared the general societal misconception that PC is end-of-life care and involves relatively straightforward, protocol-driven pharmacological treatment of symptoms. Some even equated PC with euthanasia.

Students identified quality of life as central to palliative care; “a way of improving the patient’s life” in alignment with patient and family wishes and best when initiated early in the course of chronic illness. They came to appreciate that palliative care is not just about end-of- life care. Students expressed a heightened appreciation of the value and role of palliative care and its applications in medicine in general. One student, for example, commented “Palliative [care] should be a close attitude towards life.” In fact, they viewed palliative care as a way to enhance wellbeing in the context of serious illness.

The students saw palliative care as assisting wholistic, multi-dimensional person-focused care. They came to realize that it can occur alongside treatments to control or even cure life threatening illnesses. This was “new news” for some who viewed palliative care as passive rather than active and marked an important shift in perspective away from the idea that when there are no further disease altering treatments “nothing more can be done.”

They came to understand that promoting patient wellbeing through palliative care includes preserving hope and dignity. Palliative care values “not only (patients´) physical needs but also their needs as people made up of many different dimensions.”

The students highlighted the importance of seeing things through the eyes of the patient and family, which, in their view, requires compassion, listening and dialogue.

*I will [engage] them more; not just about their condition and about possible treatment options but about what they’re feeling, their inner motivations and how they think I could care for them better. Generally to involve myself more in the human aspect of their care.*

*It’s about reasoning…maybe for one patient you choose one thing and for another patient who might be in a similar position but who has other things in their life you might decide something else. So you have to think specifically in each case what you would do and you have to reason in each of the cases*.

Students were able to see the reciprocity between their own humanity and the humanity of their patients.

*We live in a rush and we work non-stop, patients become cases and thereby we become machines. So before this could happen I will always keep in mind the fact of listening. I have learnt that many times [it] is more useful than any anatomy book. The ability of listening gives us humanity and we get closer to the patient and so to the illness course.*

*Listen to patients in a reflexive and active manner, with great empathy for them to feel accompanied. It is very important in medical practice and it will make us grow not only as physicians but also as human beings.*

Further, they now saw listening as a therapeutic intervention in and of itself. Students indicated enhanced acceptance that, in the complex world of living while dying, there may be times that they do not know what to say and that “simply listening” may be the very best thing one can do for the patient.

The course successfully challenged the notion that there is a formulaic, straightforward decision-making process in palliative care (e.g., several students commented specifically about this as it relates to artificial hydration and nutrition at end of life). Instead, it involves individualized, personalized solutions to problems that are uniquely defined by the particular circumstances, values, and desires of both patient and family, which may be different. Decision-making was understood to be more ambiguous than the students realized; taking into account multiple factors, and perspectives that requires the physician to engage in dialogue and reflection from a position of self-awareness. They became aware that there may be no clear right and wrong answers.

*I’ll consciously try to be more self-aware because during the course…I’ve realized that the way I originally thought of prospective patients and what I would do in different situations…differs from what I would wish from my [caregivers] if I were in the same position*.

*We stop being good physicians when we start treating the patient as a room number or as a research object. A good physician must know the patient as a person.*

Many students applied what they learned to practicing medicine in general – not just practicing palliative care. They saw palliative care as applicable to all physicians.

*As physicians we must be advisors and companions in the way of the illness of our patients.*

Students indicated that listening and dialogue were practices that enable better understanding of patient and family values, wishes, and decisions, which supports the physician to make better decisions. Students gained appreciation of the value of multiple perspectives, exemplified in a team approach. In particular they remarked on the importance of asking for help and of having an “open-mind.” They saw teamwork as critical to best care and decision-making. As one student noted:

*When taking a decision, no matter the importance that it can have for me, always consult with the group of people I will be working with, but also with the family and the patient [himself].*

The course invited many students to reconsider their ideas about the practice of medicine, and the role of the physician. For example, one student remarked on the “power of information” and the importance of “not opt[ing] for the easiest solutions because I am afraid of speaking truthfully with the patient.” Other students commented on their new appreciation of the importance of getting close to patients and families rather than practicing from a distance through the lens of disease and problems. The shift in perspective is further illustrated in another student’s ideas about death.

*We are taught to be doctors to save lives so it is normal to think of death as a failure but I just realized that everyone dies, earlier or not, so I should stop seeing patients like machines to [be] fix[ed] and start seeing patients like persons who deserve a better way of life.*

Another student noted that much can be done when disease oriented treatment is no longer effective but comfort in the dying process is the focus.

*Previously, before attending the palliative care course, I thought that this was a very sad specialty. Now it seems to me that it can be a very gratifying way of life – to help people in the most important moment of their lives.*

#### What difference (if any) did it make to have the course in the museum and to integrate art?

There were multiple aspects of how this course was taught that created an excellent learning experience from the students’ perspective. It was taught in a different building than “where you take exams.” The building, a museum, was spacious and offered freedom to think and move. It helped them “think in a different way; feel in a different way.” The timing of the course was June, outside the usual timetable, which again offered students time to prioritize this learning, and time to think. The students believed that, had it been offered during regular class time alongside all the other courses they needed to learn, it would have suffered because it would not have been a priority.

Students from across various years of the medical program joined to take the course. While this was a bit “intimidating” for the first-year students, they endorsed this approach because they learned so much from their classmates. They also appreciated early introduction of palliative care in their learning.

The way the course was taught was completely different than the theoretical approach taken in other classes. This class was practical and case based; requiring understanding and reasoning prior to decision making.

The students were clear that integrating art in the learning experience assisted them in one of two ways. For some students, art directly influenced their learning; whereas, for others, it was the discussion about the art that was influential. The students believed it enabled them to both see and appreciate the validity and usefulness of multiple perspectives and the importance of observation. Students who found the art directly helpful explained it this way:

*I also liked the way the teacher tried to compare a picture that they just explained to us, and tried to compare that to the real patient or the situation in that moment. So for me it was really useful trying to imagine that picture and trying to comparing with that situation.*

*I think that to be a good physician you have to be very sensitive to what happens around you and that art helps to see and promote that sensitivity; to be aware of things that are happening around you.*

Another student experienced art as a way of connecting with and understanding self.

*There were different rooms of art, there’s one abstract and there’s another one where there’s mountains downstairs. I made different connections with myself. I like going to mountains, for me it’s different, the emotions I felt were quite different seeing the different pieces of art.*

Art also offered a different way of knowing, feeling and expressing than can be captured in words, mirroring the practice of PC.

*We have learned the necessity of reflecting about intrinsic questions of the human being. For example, dignity and hope. In the process of expressing ourselves in the museum in the art, it became clear that words alone are not able to express things fully.*

In addition, art enabled the growth of the human being who was becoming a physician.

*Also I think as a doctor, as a student, we should know about art, we should know about literature, we have to learn about everything. This is a good way to learn art in my opinion.*

Other students did not find the art helpful. But the discussion about the art provided a unique learning opportunity. It enabled empathy and facilitated openness to multiple perspectives.

*I can’t really see what other people might see. But for me what was more important was to try and understand what other people see and how they see it. And try to get into someone else’s head because it’s not all the same for everyone. I see the red picture and that’s it. And if someone else says, ‘I feel whatever when I see that picture,’ I think about it and I try to understand why they see that and where they are coming from.*

*I don’t know anything about art but I like that we had to put on the other’s shoes. I think that every doctor should put on the patient’s shoes. So I like that relation.*

Finally, the students offered ideas about how to make this course even better. They appreciated the small group setting and the intimacy of the discussions. They would have liked to get to know one another better at the beginning of the course, and to engage in deeper discussion in even smaller groups, sitting in a circle, on the same level, seeing each other’s faces.

In summary, the students deeply appreciated the coherence of the learning experience, which aligned with the nature of palliative care. It was a learning experience that engaged them as whole persons learning to care authentically with and for whole persons in the vulnerable situation of advanced illness.

## Discussion

This course, in addition to introducing medical students from different years to the principles of palliative care, also exposed them to essential attitudes and behaviours that contribute to wholistic, shared control, patient-centred care [17]. The students learned about palliative and end-of-life care, about themselves, and about how they would like to practice in general. Their understanding of palliative care changed and misconceptions were dispelled. They came to understand that palliative care is not restricted to the end-of-life (terminal) phase of illness, that it represents active care and that it can be done alongside treatments to control or cure the disease. They came to appreciate the multifaceted nature of decision-making in the palliative care setting and the need to individualize care plans. Palliative care therefore served as a vehicle to address several competencies, not only those related to caring for persons with life threatening illnesses.

Moreover, the course resulted in a re-conceptualization of relationships with patients and families, as well as the role of the physician in palliative and end of life care decision-making. For some, there was a broader reconceptualization of the practice of medicine and a greater appreciation of the components that contribute to empathy, where *empathy* refers to one’s ability to experience the feelings, thoughts, viewpoints and values of another [6]. Arts (performing, literary and visual) and the humanities have previously been included, alongside other approaches such as narrative reflections, in methods to introduce and nurture empathy in medical education [18–20].

The use of the visual arts for teaching palliative care is still an uncharted territory. Turton et al. in a recent scoping review described that visual arts, employed as a method for training palliative care professionals, improves the awareness of others, personal development and self-awareness [21]. Our study was not designed to assess the specific contribution of the arts relative to the other course components. There is however evidence from our course that the use of the visual arts and the setting (in modern art museum) enhanced the learning experience for many of the students. In particular, students came to realize that the same piece of art could be seen and interpreted in different ways by different individuals. Harnessing the arts and humanities to facilitate palliative care education has previously been reported [12–14, 22]. Johnson and Jackson, for example, have described the contributions of the arts and humanities as teaching and learning strategies to support palliative care education [14]. Lawton and McKie reported a staff seminar programme that used art and literature as vehicles to explore personal and professional dimensions of palliative care [12]. A variety of media, including the visual arts, were used to facilitate learners’ expressions of their learning reflections [12].

Benefits of incorporating the arts and humanities in general medical education have been reported. They can, it has been argued, facilitate the acquisition of a variety of competencies that range from empathy and cultural sensitivity to clinical observation and diagnostic skills [23–30]. Zazuluk et al. paired family medicine residents with an art educator and a family physician and required the residents to undertake reflective practice exercises with the goal of improving empathy amongst the learners as well as enhancing their observational skills [18]. Schaff and colleagues explain that art interpretation and daily clinical work share some similarities such as the importance of observation, multiple interpretations, ambiguity, collaboration between observers and direction to action [31].

Debate continues as to the evidence for incorporating the arts and humanities in medical education. Ousager and Johannessen, for example, have posited that, based on their systematic review, evidence on the positive long-term impacts of integrating humanities into undergraduate medical education is sparse [32]. This, they go on to say, “may pose a threat to the continued development of humanities-related activities in undergraduate medical education in the context of current demands for evidence to demonstrate educational effectiveness.” Belling, on the other hand, contended “the value of the humanities in educating new physicians can be defended by demonstrating the need for more complex approaches to knowledge than complete dependence on empirical evidence” [33].

This course used a combination of approaches to achieve the learning objectives. These included the visual arts, case studies, stories (narratives by faculty), reflective trigger videos, group discussions and an interactive open milieu. While learners specifically reported the impact of incorporating the arts and the uniqueness of the setting, the evaluation framework used was not designed to systematically tease out the relative and specific contributions of each of the different methods. The course and its impact on learners therefore need to be viewed collectively as a package of different approaches.

Several limitations are acknowledged, particularly with respect to the generalizability of the course design. Firstly, the students were a group of highly motivated, self-identified group of individuals; they volunteered to participate outside their usual class-time. They were open to the experience and the learning methods, including the arts, used. Secondly, the course was delivered outside the academic year during the beginning of summer holidays. It can therefore be argued that it may be difficult to incorporate such a class in an already full curriculum during normal academic hours.

Given the relatively small number of learners, we were not able to compare the experiences of students who are in the pre-clinical years versus those in the clinical years. The impact of experiential learning with patients to augment this course merits future attention. The imbalance between female and male students can be largely explained by that fact that in the university of Navarre, between 70 and 80% of medical students are currently women.

Strategies could be developed to incorporate this course in the second or third years. With 180 students per year in the Faculty of Medicine at the University of Navarra, it is feasible to hold the course four or five times a year during the academic year (in the evenings), thereby maintaining the intimacy and reflective learning potentials of small learning in this unique setting (modern art museum). More opportunities should be sought to incorporate palliative care education and nurturing wholistic, patient-centred and empathic care throughout the six-year curriculum. While the arts are not for everyone, as was reported by some of the students who participated in this pilot course, even these students recognized that it opened their minds and perspectives.

This course could be adapted for an interprofessional audience. It would require some redesigning so that it addreses the competences that are common to all participating professions while also addresing the competencies that are unique to each of the professions. Learning these topics has the capacity of enhancing team work.

## Conclusions

In this pilot course of 20 h delivered to medical students from the second to the sixth years of medicine, students learned about palliative and end of life care, about themselves, and about how they would like to practice in general. Their understanding of palliative care changed and misconceptions were dispelled. As a main learning objective, they came to appreciate the multifaceted nature of decision-making in the palliative care setting and the need to individualize care plans. Moreover, the course resulted in a re-conceptualization of relationships with patients and families, as well as their role as future physicians. Palliative care decision-making therefore, augmented by the visual arts, can serve as a vehicle to address several competencies, including those related to being patient-centred, empathic and wholistic in the approach to care. These can be incorporated and re-emphasized longitudinally across the whole medical curriculum, from the pre-clinical to the clinical years of medical school, and beyond into specialization programs. The specific contributions of the different learning methods used in this course, including the arts, needs further study.

## Abbreviations

AN: Antonio Noguera
CC: Carlos Centeno
CR: Carole Robinson
ECTS: European Credit Transfer and Accumulation System
ICS: Institute of Culture and Society (ICS)
JP: Jose Pereira
LEAP: Learning Essential Approaches to Palliative Care
MA: Maria Arantzamendi
PC: Palliative Care
WHO: World Health Organization
